# Supplementary material for: Nuclear membrane protein Lem2 regulates nuclear size through membrane flow
Source: Nat Commun. 2019 Apr 23;10:1871. doi: 10.1038/s41467-019-09623-x (PMC6478680; doi:10.1038/s41467-019-09623-x)
Supplement: Supplementary file 1 — Supplementary Information [file 41467_2019_9623_MOESM1_ESM.pdf]

Nuclear membrane protein Lem2 regulates nuclear size through membrane flow

Kume *et. al.*

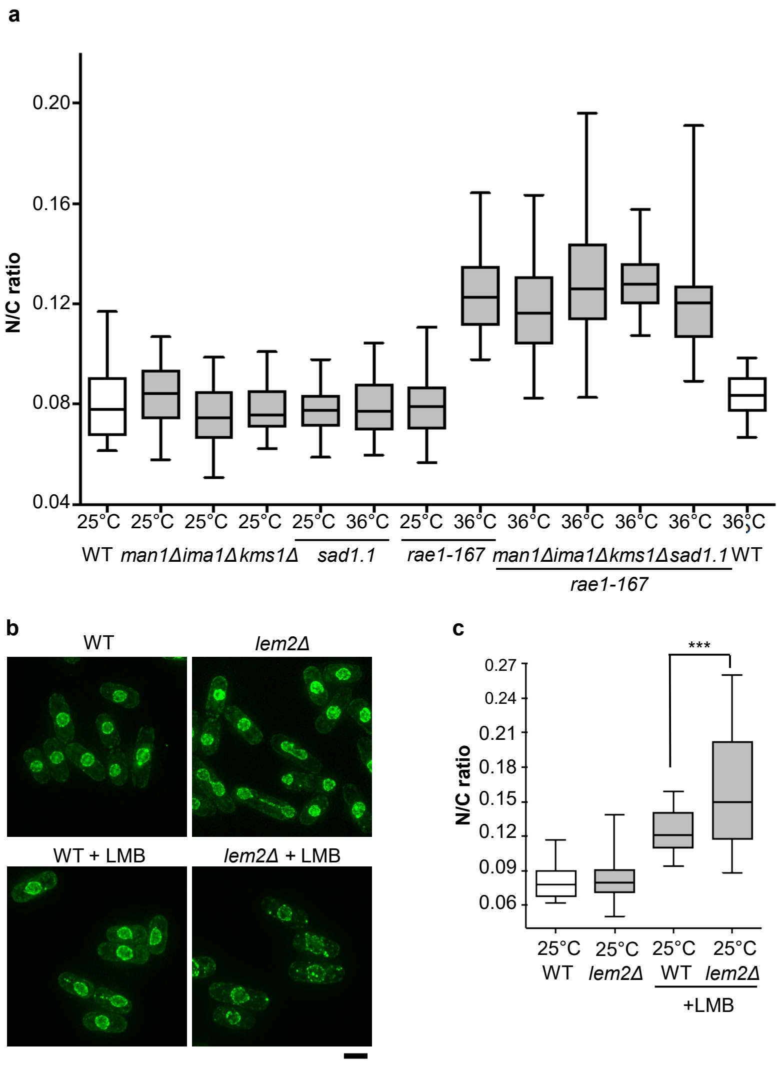

Supplementary Figure 1: Lem2 deletion enhances nuclear size enlargement

(a) N/C ratio of wild type (WT), *man1*Δ, *ima1*Δ, *kms1*Δ, *sad1.1*, *rae1-167*, *rae1-167 man1*Δ, *rae1-167 ima1*Δ, *rae1-167 kms1*Δ and *rae1-167 sad1.1* cells (25˚C) shifted to the indicated temperature for four hours (n > 30 cells). Two-tailed Mann Whitney tests: *man1*Δ (n = 43 cells), *ima1*Δ (n = 36 cells), *kms1*Δ (n = 32 cells) and *sad1.1* (36˚C) (n = 31 cells) against WT (n = 35 cells), *P* = 0.1574, 0.1530, 0.7695 and 0.0761 respectively; *rae1-167 man1*Δ (n = 34 cells), *rae1-167 ima1*Δ (n = 42 cells), *rae1-167 kms1*Δ (n = 31 cells) and *rae1-167 sad1.1* (n = 35 cells) against *rae1-167* (n = 43 cells), *P* = 0.1282, 0.3742, 0.1609 and 0.2217 respectively. The corresponding dot plot is available in Supplementary Fig. 10a.

(b) Images of the nuclear envelope (Cut11-GFP, green) of WT and *lem2*Δ cells (25˚C) untreated or treated with 100 ng/ml leptomycin B (+ LMB) for three hours. Maximum intensity projections shown. Scale bar: 5 µm.

(c) N/C ratio of WT and *lem2*Δ cells (25˚C) untreated or treated with 100 ng/ml leptomycin B (+LMB) for three hours (n > 30 cells). Two-tailed Mann Whitney tests: ***P* = 0.0013, WT + LMB (n = 32 cells) against *lem2*Δ + LMB (n = 32 cells); *lem2*Δ (n = 52 cells) against WT (n = 35 cells), *P* = 0.8062. The corresponding dot plot is available in Supplementary Fig. 10b.

In all box-and-whiskers diagrams, boxes indicate median and upper and lower quartile and whiskers indicate range of data.

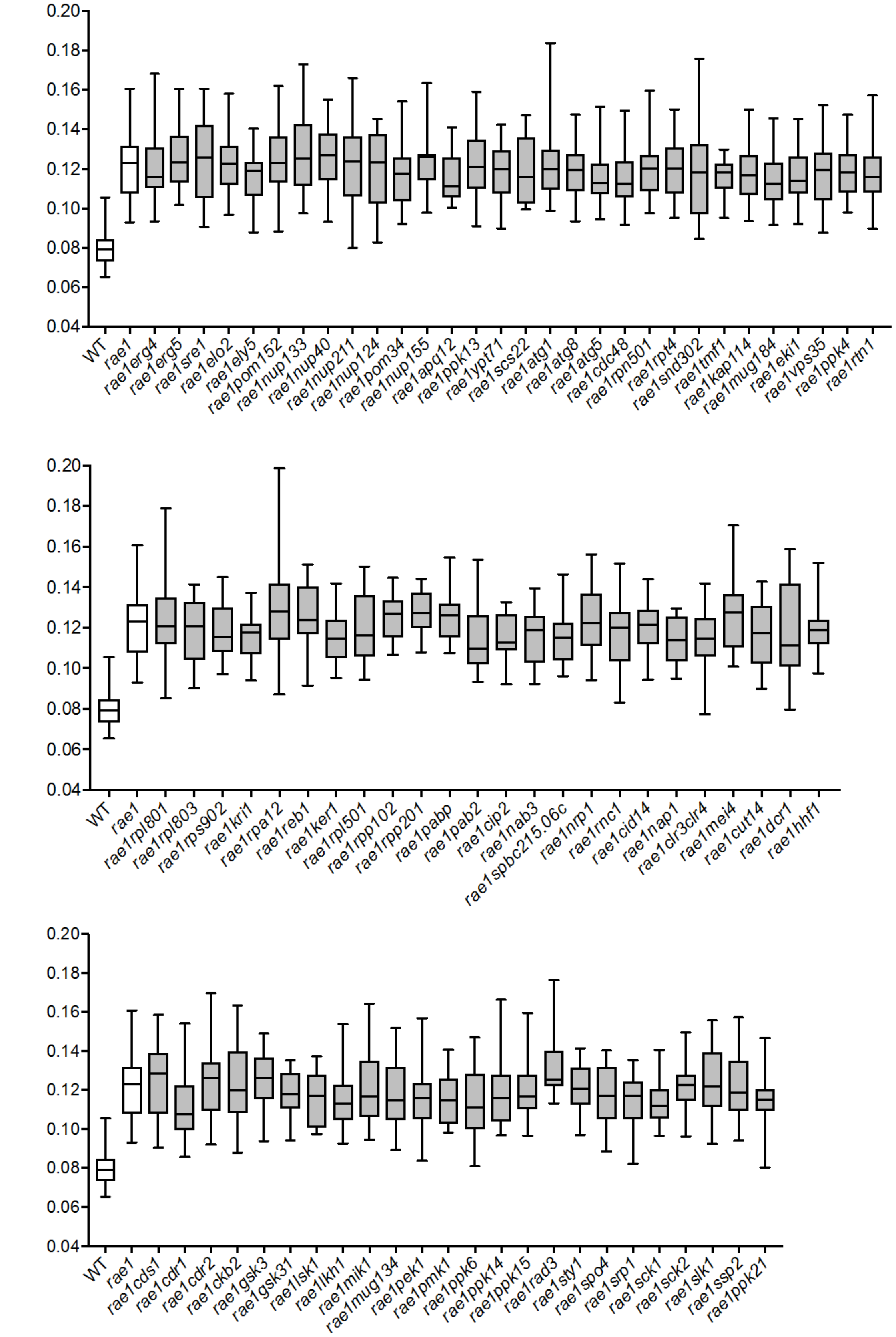

Supplementary Figure 2: Double mutant analysis of *rae1-167* with proteins reported to localise to the nucleus, the endoplasmic reticulum and /or other organelles

N/C ratio of WT, *rae1-167* and indicated double mutants. Cells grown at 25˚C were shifted to 36˚C for four hours (n ≧ 20 cells). In box-and-whiskers diagrams, boxes indicate median and upper and lower quartile and whiskers indicate range of data.

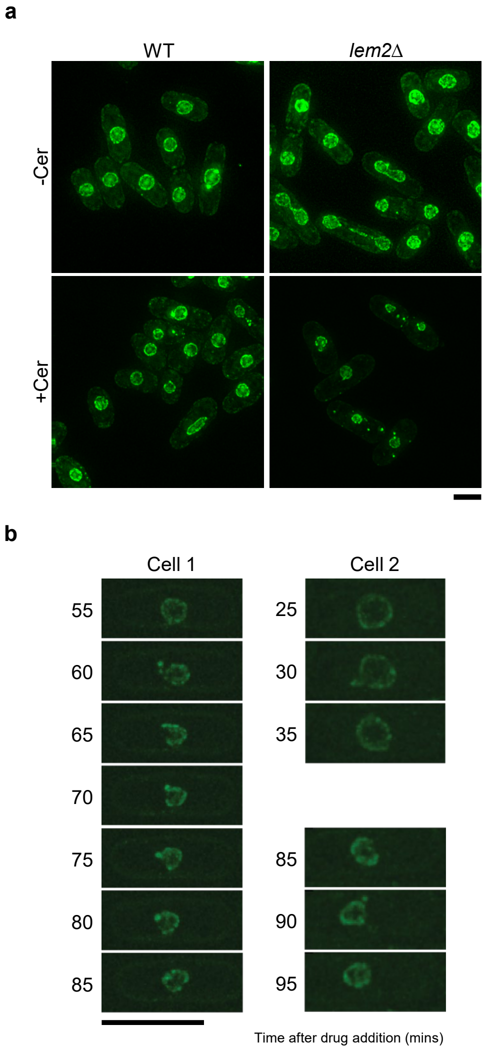

Supplementary Figure 3: Cerulenin treatment causes shrinkage of interphase *lem2*Δ but not wild type nuclei
(a) Images of the nuclear envelope (Cut11-GFP, green) of wild type (WT) and *lem2*Δ cells grown at 25˚C untreated (-Cer) or treated (+Cer) with 10 µg/ml cerulenin for three hours. Maximum intensity projections shown. Scale bar: 5 µm.
(b) Stills from timelapse imaging of *lem2*Δ cells treated with 10 µg/ml cerulenin at 32˚C that show blebbing. Time after drug addition indicated. Scale bar: 10 µm. Nuclear envelope (Cut11-GFP, green) maximum intensity projection.

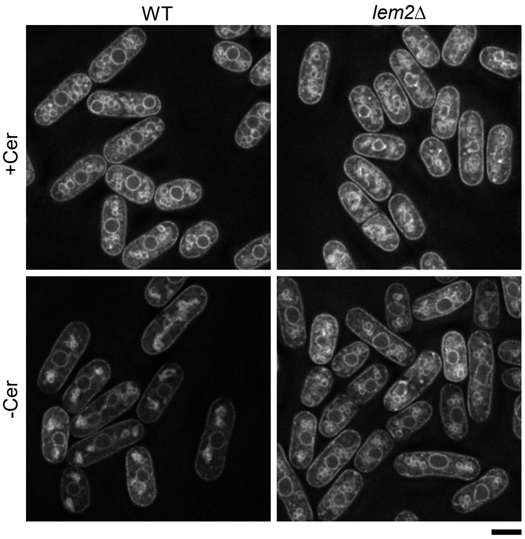

Supplementary Figure 4: Membrane staining indicates aberrant nuclear shape and membrane foci on nuclear envelope of *lem2*Δ cells treated with cerulenin

Images of wild type (WT) and *lem2*Δ cells, untreated (-Cer) and following 90 mins of treatment with 10 µg/ml cerulenin (+Cer) at 32˚C. Cellular membranes visualized by $DiOC_6$ staining. Single z-slice shown. Scale bar: 5 µm.

a

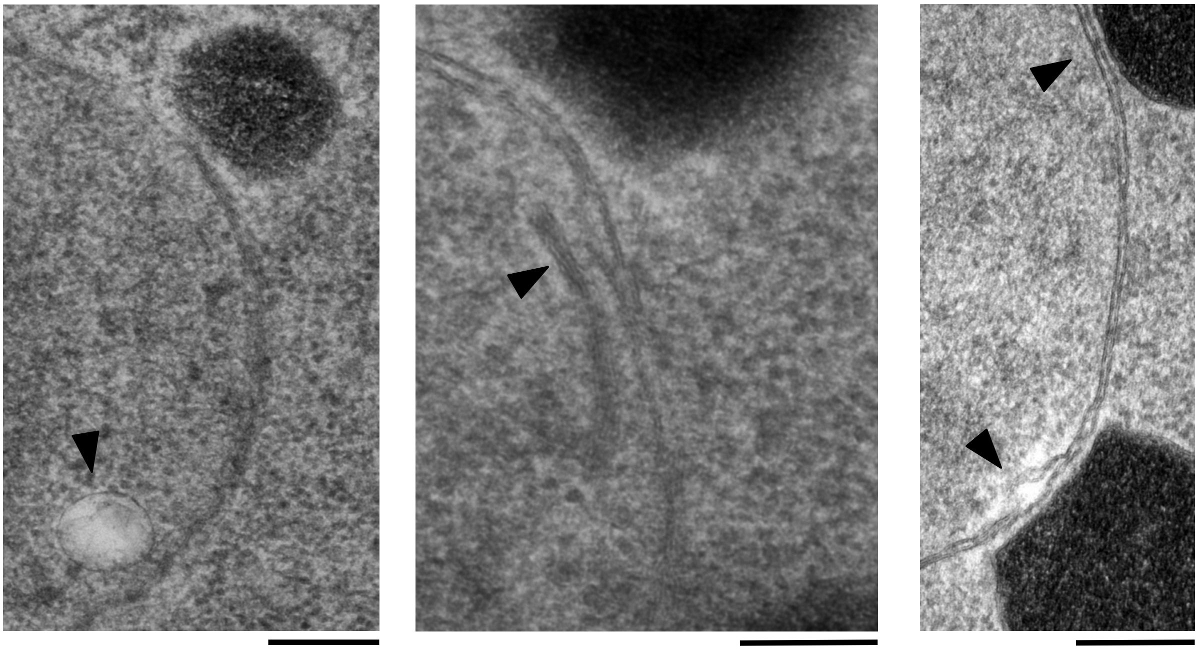

b

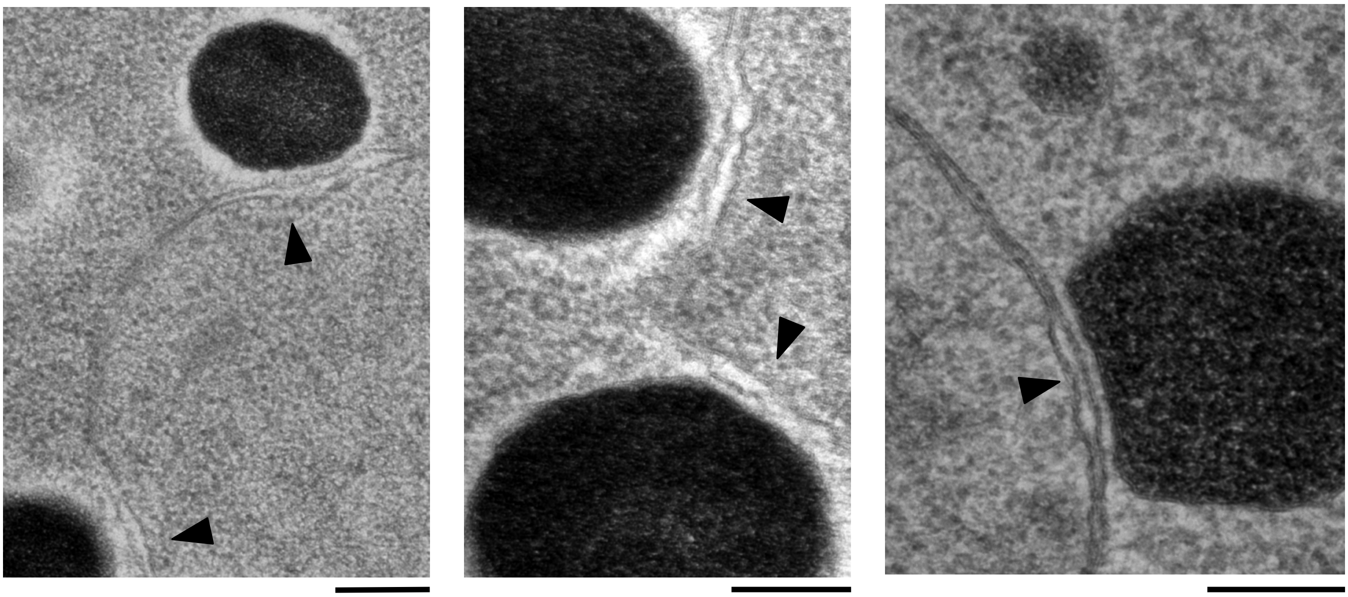

Supplementary Figure 5: Membranous compartments in close proximity to the nuclear envelope and intranuclear membranous structures are visible in cerulenin treated *lem2*Δ cells.
Transmission electron microscopy images of *lem2*Δ cells treated with 10 μg/ml cerulenin for 90 minutes displaying intranuclear membranous structures (a) and membranous compartments in close proximity to the nuclear envelope (b) indicated by black arrowheads. Scale bars: 250 nm.

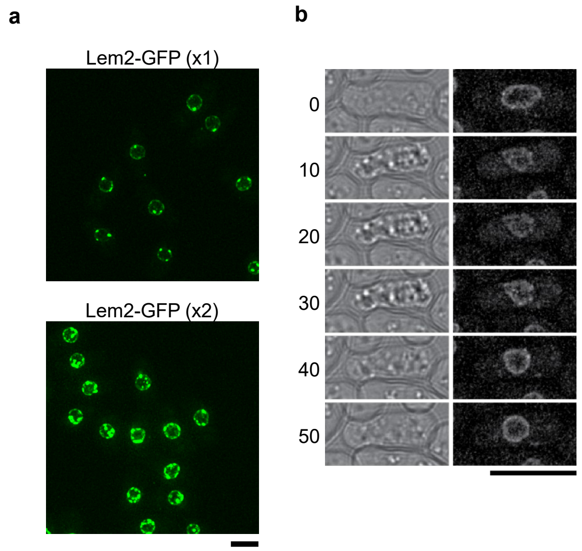

Supplementary Figure 6: Lem2 localisation is not affected by increasing its copy number

(a) Images of wild type cells grown at 25˚C expressing 1 copy or 2 copies of Lem2-GFP (Lem2-GFP, green). Single z-slice shown. Scale bar: 5 µm.

(b) Stills from timelapse microscopy of a *lem2*Δ cell grown at 25˚C (EtOH treated as control) displaying nuclear shrinkage. Time in minutes indicated. Scale bar: 10 µm. Cell (brightfield, grayscale, left hand column), nuclear envelope maximum intensity projection (Cut11-GFP, grayscale, right hand column).

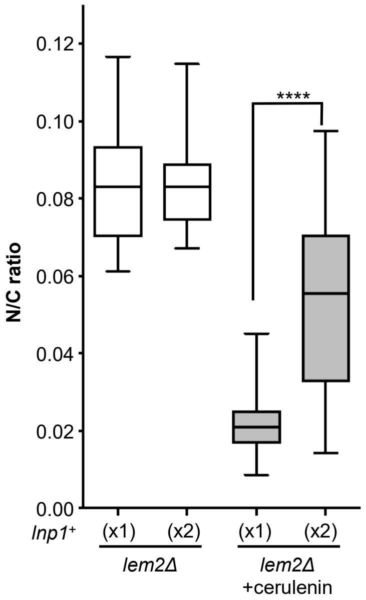

Supplementary Figure 7: Increased copy number of lnp1 suppresses the reduction of N/C ratio caused by cerulenin treatment of *lem2*Δ cells

N/C ratio of *lem2*Δ cells containing one (x1) or two (x2) copies of the gene encoding Lnp1 grown at 25˚C untreated or treated with 10 µg/ml cerulenin (+Cer) for three hours. (n > 30 cells). Two-tailed Mann Whitney test: ****$P <$ 0.0001, *lem2*Δ + 1x*lnp1*$^{+}$ + cerulenin (n = 32 cells) against *lem2*Δ + 2x*lnp1*$^{+}$ + cerulenin (n = 33 cells). In box-and-whiskers diagrams, boxes indicate median and upper and lower quartile and whiskers indicate range of data. The corresponding dot plot is available in Supplementary Fig. 10c.

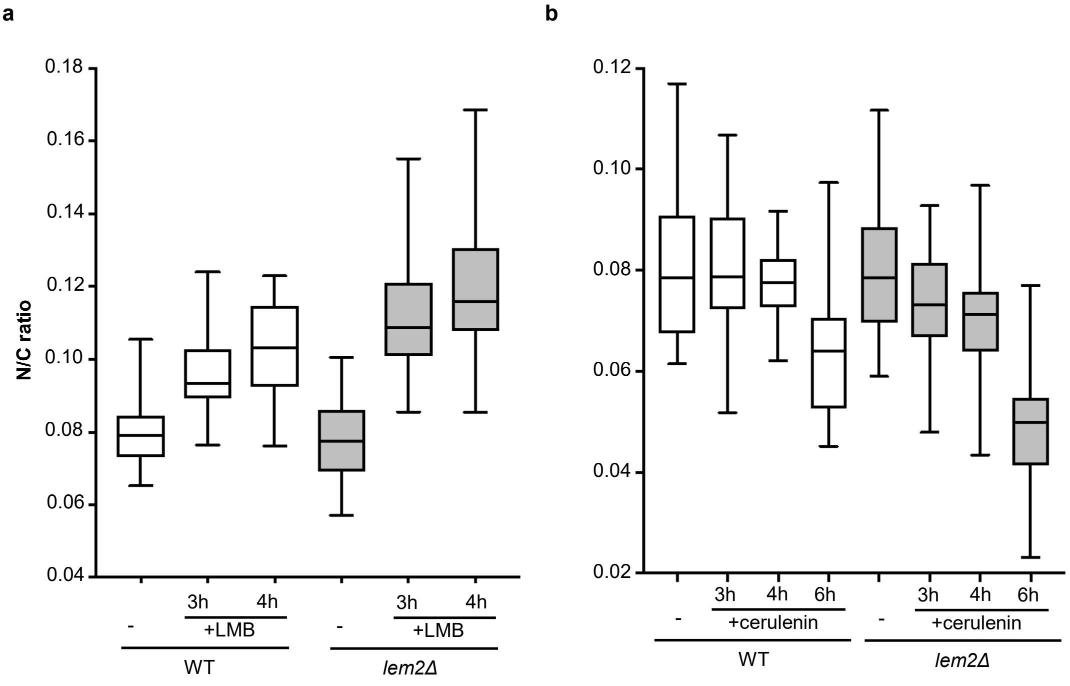

Supplementary Figure 8: The nuclear size phenotypes of *lem2* deletion cells grown in EMM medium
(a) N/C ratio of wild type (WT), and *lem2*Δ cells grown in EMM medium with or without 100 ng/ml leptomycin B (+LMB) for three hours or four hours (25˚C) (n > 22 cells). The corresponding dot plot is available in Supplementary Fig. 10d.
(b) N/C ratio of WT and *lem2*Δ cells grown in EMM medium with or without 10 µg/ml cerulenin for three, four or six hours (25˚C) (n > 30 cells). The corresponding dot plot is available in Supplementary Fig. 10e.
In all box-and-whiskers diagrams, boxes indicate median and upper and lower quartile and whiskers indicate range of data.

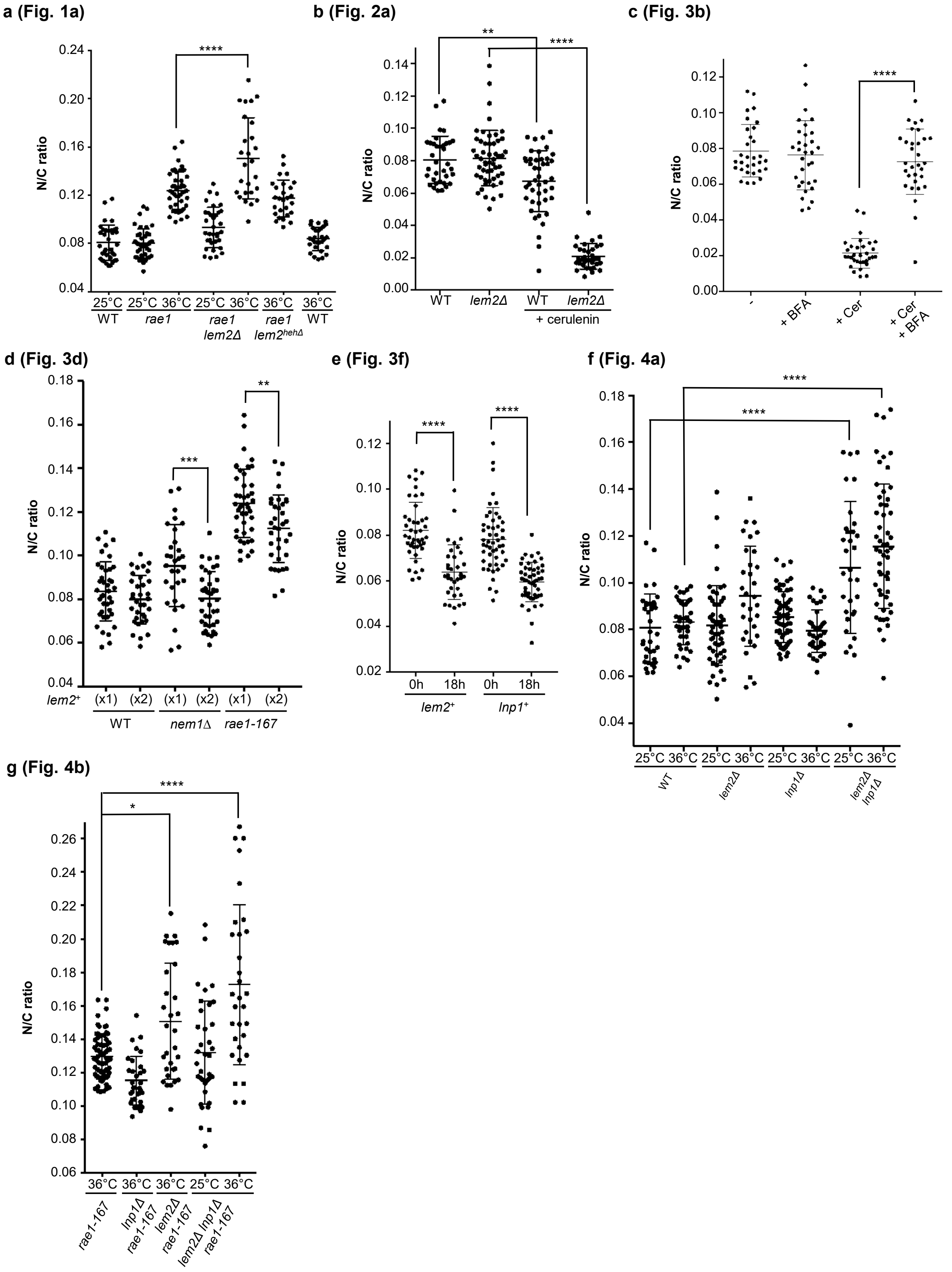

Supplementary Figure 9: Dot plot of N/C ratios shown in the indicated Figures
Error bars represent mean ± s.d.

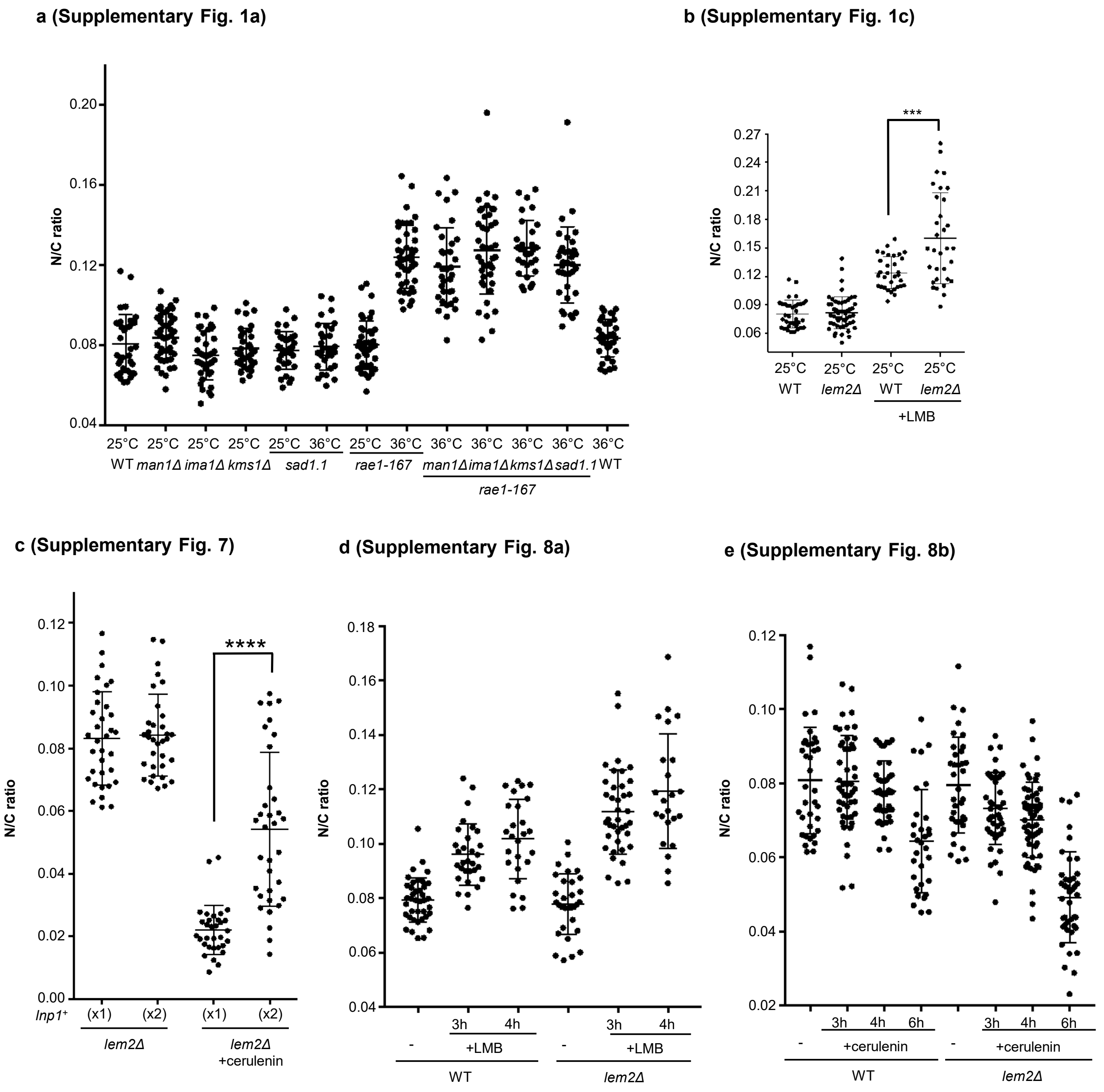

Supplementary Figure 10: Dot plot of N/C ratios shown in the indicated Supplementary Figures
Error bars represent mean ± s.d.

Supplementary Table 1: *S. pombe* strains used in this study

Strain	Genotype	Sources	
PN10345	h$^-$ cut11$^+$:GFP:ura4$^+$ ura4-D18	*Neumann & Nurse 2007*	Fig.1a, 1b, 2a-c, 2g, 3c, 3d, 4a, 4c, Supplementary Fig.1a-c, 2, 3a, 8, Supplementary Movie 1-4
UKK412	h$^-$ rae1-167 cut11$^+$:GFP:ura4$^+$ ura4-D18 leu1-32 ade6	*Kume et al.* 2017	Fig.1a, 1b, 3d, 4b, 4c, Supplementary Fig.1a, 2
UKK737-2C	h$^-$ rae1-167 lem2Δ::kan^r cut11$^+$:GFP:ura4$^+$ ura4-D18 leu1-32 ade6	This study	Fig.1a, 1b, 4b, 4c
UKK925-5B	h$^?$ rae1-167 lem2Δ::kan^r cut11$^+$:GFP:ura4$^+$ leu1-32:lem2$^{heh\Delta}$ ura4-D18 ade6	This study	Fig.1a
UKK794-1A	h$^?$ rae1-167 lem2Δ::kan^r cut11$^+$:GFP:ura4$^+$ hht1$^+$:mRFP:kan^r ura4-D18 leu1-32 ade6	This study	Fig.1c
UKK737-4A	h$^-$ lem2Δ::kan^r cut11$^+$:GFP:ura4$^+$ ura4-D18 leu1-32 ade6	This study	Fig. 2a-d, 2g, 2h, 3a-d, 4a, 4c, Supplementary Fig.1b, 1c, 3a, 5b, 6b, 7, 8, and Supplementary Movie 1-4
PN5603	h$^?$ sid4$^+$:mRFP:kan^r cut11$^+$:GFP:ura4$^+$ ura4-D18 leu1-32 ade6	This study	Fig. 2e, Supplementary Fig.3b, Supplementary Movie 5-8
PN5602	h$^?$ lem2Δ::kan^r sid4$^+$:mRFP:kan^r cut11$^+$:GFP:ura4$^+$ ura4-D18 leu1-32 ade6	This study	Fig. 2e, Supplementary Fig.3b, Supplementary Movie 5-8
PN5605	h$^?$ sid4$^+$:mRFP:kan^r ura4-D18 leu1-32 ade6	This study	Fig. 2f, Supplementary Fig.4
PN5604	h$^?$ lem2Δ::kan^r sid4$^+$:mRFP:kan^r ura4-D18 leu1-32 ade6	This study	Fig. 2f, Supplementary Fig.4
UKK342-1A	h$^-$ nem1Δ::kan^r cut11$^+$:GFP:ura4$^+$ ura4-D18 leu1-32 ade6	*Kume et al.* 2017	Fig. 3c, 3d
UKK842-1B	h$^?$ cut11$^+$:GFP:ura4$^+$ leu1-32:lem2$^+$:leu1$^+$ ura4-D18	This study	Fig. 3c, 3d
UKK843-2A	h$^?$ nem1Δ::kan^r cut11$^+$:GFP:ura4$^+$ leu1-32:lem2$^+$:leu1$^+$ elo2$^+$:mCherry:nat^r ura4-D18	This study	Fig. 3c, 3d
UKK843-1A	h$^?$ rae1-167 cut11$^+$:GFP:ura4$^+$ leu1-32:lem2$^+$:leu1$^+$ ura4-D18	This study	Fig. 3d
UKK2214-3D	h$^?$ cut11$^+$:mCherry:nat^r pBip1:GFP:ADEL:ura4$^+$ ura4-D18 leu1-32	This study	Fig. 3e
UKK2210-10B	h$^?$ nem1Δ::kan^r cut11$^+$:mCherry:nat^r pBip1:GFP:ADEL:ura4$^+$ ura4-D18 leu1-32 ade6	This study	Fig. 3e
UKK2210-1C	h$^?$ nem1Δ::kan^r cut11$^+$:mCherry:nat^r pBip1:GFP:ADEL:ura4$^+$ leu1-32:lem2$^+$:leu1$^+$ ura4-D18 ade6	This study	Fig. 3e
UKK2255-3B	h$^?$ nem1Δ::kan^r cut11$^+$:mCherry:nat^r pBip1:GFP:ADEL:ura4$^+$ leu1-32:lnp1$^+$:leu1$^+$ ura4-D18	This study	Fig. 3e
UKK2212	h$^-$ kan^r:nmt1:GFP:lem2$^+$ cut11$^+$:mCherry:nat^r ura4-D18 leu1-32	This study	Fig. 3f
UKK2215	h$^-$ kan^r:nmt1:GFP:lnp1$^+$ cut11$^+$:mCherry:nat^r ura4-D18 leu1-32	This study	Fig. 3f
UKK1708-3A	h$^?$ lnp1Δ::kan^r cut11$^+$:GFP:ura4$^+$ ura4-D18 leu1-32 ade6	This study	Fig. 4a, 4c
UKK1867-1A	h$^?$ lem2Δ::kan^r lnp1Δ::kan^r cut11$^+$:GFP:ura4$^+$ ura4-D18 ade6	This study	Fig. 4a, 4c
UKK1708-1B	h$^?$ rae1-167 lnp1Δ::kan^r cut11$^+$:GFP:ura4$^+$ ura4-D18 leu1-32 ade6	This study	Fig. 4b, 4c

UKK1879-1C	*h? rae1-167 lem2Δ::kan^r lnp1Δ::kan^r cut11^+:GFP:ura4^+ ura4-D18 ade6*	This study	Fig. 4b, 4c
UKK463-1B	*h^- man1Δ::kan^r cut11^+:GFP:ura4^+ ura4-D18 ade6*	This study	Supplementary Fig.1a
UKK546-2C	*h? rae1-167 man1Δ::kan^r cut11^+:GFP:ura4^+ leu1-32 ura4-D18 ade6*	This study	Supplementary Fig.1a
UKK986-1A	*h? ima1Δ::kan^r cut11^+:GFP:ura4^+ leu1-32 ura4-D18 ade6*	This study	Supplementary Fig.1a
UKK986-2C	*h? rae1-167 ima1Δ::kan^r cut11^+:GFP:ura4^+ leu1-32 ura4-D18 ade6*	This study	Supplementary Fig.1a
UKK985-2A	*h? kms1Δ::kan^r cut11^+:GFP:ura4^+ leu1-32 ura4-D18 ade6*	This study	Supplementary Fig.1a
UKK985-1B	*h? rae1-167 kms1Δ::kan^r cut11^+:GFP:ura4^+ leu1-32 ura4-D18 ade6*	This study	Supplementary Fig.1a
UKK1060-4C	*h^- sad1.1 cut11^+:GFP:ura4^+ ura4-D18 leu1-32*	This study	Supplementary Fig.1a
UKK1846-1B	*h^- rae1-167 sad1.1 cut11^+:GFP:ura4^+ ura4-D18 leu1-32*	This study	Supplementary Fig.1a
UKK1806-3A	*h^- rae1-167 erg4Δ::ura4^+ cut11^+:mCherry:nat^r ura4-D18 leu1-32*	This study	Supplementary Fig.2
UKK542-1C	*h? rae1-167 erg5Δ::kan^r cut11^+:GFP:ura4^+ ura4-D18 leu1-32 ade6*	This study	Supplementary Fig.2
UKK543-2B	*h? rae1-167 sre1Δ::kan^r cut11^+:GFP:ura4^+ ura4-D18 leu1-32 ade6*	This study	Supplementary Fig.2
UKK1827-1D	*h? rae1-167 elo2Δ::kan^r cut11^+:GFP:ura4^+ ura4-D18 leu1-32 ade6*	This study	Supplementary Fig.2
UKK708-1D	*h? rae1-167 ely5Δ::kan^r cut11^+:GFP:ura4^+ ura4-D18 leu1-32 ade6*	This study	Supplementary Fig.2
UKK818-1B	*h? rae1-167 pom152Δ::kan^r cut11^+:GFP:ura4^+ ura4-D18 leu1-32 ade6*	This study	Supplementary Fig.2
UKK1046-6B	*h? rae1-167 nup133Δ::kan^r cut11^+:GFP:ura4^+ ura4-D18 leu1-32 ade6*	This study	Supplementary Fig.2
UKK1000-1D	*h? rae1-167 nup40Δ::kan^r cut11^+:GFP:ura4^+ ura4-D18 leu1-32 ade6*	This study	Supplementary Fig.2
UKK1136-3D	*h? rae1-167 nup211Δ::kan^r cut11^+:GFP:ura4^+ ura4-D18 leu1-32 ade6*	This study	Supplementary Fig.2
UKK1313-5B	*h? rae1-167 nup124Δ::kan^r cut11^+:GFP:ura4^+ ura4-D18 leu1-32 ade6*	This study	Supplementary Fig.2
UKK2257-3A	*h^+ rae1-167 pom34Δ::kan^r cut11^+:GFP:ura4^+ ura4-D18 leu1-32*	This study	Supplementary Fig.2
UKK2256-2B	*h? rae1-167 nup155Δ::kan^r cut11^+:GFP:ura4^+ ura4-D18 leu1-32 ade6*	This study	Supplementary Fig.2
UKK1400-1A	*h? rae1-167 apq12Δ::kan^r cut11^+:GFP:ura4^+ ura4-D18 leu1-32 ade6*	This study	Supplementary Fig.2
UKK1614-1A	*h^+ rae1-167 ppk13Δ::ura4^+ cut11^+:mCherry:nat^r ura4-D18 leu1-32 his2 ade6*	This study	Supplementary Fig.2
UKK461-1A	*h? rae1-167 ypt71Δ::kan^r cut11^+:GFP:ura4^+ ura4-D18 leu1-32*	This study	Supplementary Fig.2
UKK1160-1A	*h? rae1-167 scs22Δ::kan^r cut11^+:GFP:ura4^+ ura4-D18 leu1-32 ade6*	This study	Supplementary Fig.2
UKK1616-1C	*h^+ rae1-167 atg1Δ::ura4^+ cut11^+:mCherry:nat^r ura4-D18 leu1-32 his2*	This study	Supplementary Fig.2
UKK633-5B	*h? rae1-167 atg8Δ::kan^r cut11^+:GFP:ura4^+ ura4-D18 leu1-32 ade6*	This study	Supplementary Fig.2
UKK457-3C	*h? rae1-167 atg5Δ::kan^r cut11^+:GFP:ura4^+ ura4-D18 leu1-32 ade6*	This study	Supplementary Fig.2

UKK1407-1A	h^{-} rae1-167 cdc48-353 cut11^{+}:GFP:ura4^{+} ura4-D18? leu1-32 ade6?	This study	Supplementary Fig.2
UKK1379-3D	$h^{?}$ rae1-167 rpn501Δ::kan^{r} cut11^{+}:GFP:ura4^{+} ura4-D18 leu1-32 ade6	This study	Supplementary Fig.2
UKK1401-2B	$h^{?}$ rae1-167 rpt4Δ::kan^{r} cut11^{+}:GFP:ura4^{+} ura4-D18 leu1-32 ade6	This study	Supplementary Fig.2
UKK1408-2C	$h^{?}$ rae1-167 snd302Δ::kan^{r} cut11^{+}:GFP:ura4^{+} ura4-D18 leu1-32 ade6	This study	Supplementary Fig.2
UKK1592-7C	$h^{?}$ rae1-167 tmf1Δ::kan^{r} cut11^{+}:GFP:ura4^{+} ura4-D18 leu1-32 ade6	This study	Supplementary Fig.2
UKK1540-2C	$h^{?}$ rae1-167 kap114Δ::kan^{r} cut11^{+}:GFP:ura4^{+} ura4-D18? leu1-32	This study	Supplementary Fig.2
UKK1393-1C	$h^{?}$ rae1-167 mug184Δ::kan^{r} cut11^{+}:GFP:ura4^{+} ura4-D18 leu1-32 ade6	This study	Supplementary Fig.2
UKK1572-2A	$h^{?}$ rae1-167 eki1Δ::kan^{r} cut11^{+}:GFP:ura4^{+} ura4-D18? leu1-32 ade6	This study	Supplementary Fig.2
UKK1468-1B	$h^{?}$ rae1-167 vps35Δ::kan^{r} cut11^{+}:GFP:ura4^{+} ura4-D18? leu1-32 ade6	This study	Supplementary Fig.2
UKK1576-2A	h^{+} rae1-167 ppk4Δ::ura4^{+} cut11^{+}:mCherry:nat^{r} ura4-D18 leu1-32 his2	This study	Supplementary Fig.2
UKK2261-1A	h^{-} rae1-167 rtn1Δ::kan^{r} cut11^{+}:GFP:ura4^{+} ura4-D18 leu1-32 ade6	This study	Supplementary Fig.2
UKK686-1B	$h^{?}$ rae1-167 rpl801Δ::kan^{r} cut11^{+}:GFP:ura4^{+} ura4-D18 ade6	This study	Supplementary Fig.2
UKK1394-2A	$h^{?}$ rae1-167 rpl803Δ::kan^{r} cut11^{+}:GFP:ura4^{+} ura4-D18 leu1-32 ade6	This study	Supplementary Fig.2
UKK1294-4B	$h^{?}$ rae1-167 rps902Δ::kan^{r} cut11^{+}:GFP:ura4^{+} ura4-D18 leu1-32 ade6	This study	Supplementary Fig.2
UKK1395-2B	$h^{?}$ rae1-167 kri1Δ::kan^{r} cut11^{+}:GFP:ura4^{+} ura4-D18 leu1-32 ade6	This study	Supplementary Fig.2
UKK1597-1A	$h^{?}$ rae1-167 rpa12Δ::kan^{r} cut11^{+}:GFP:ura4^{+} ura4-D18? leu1-32 ade6	This study	Supplementary Fig.2
UKK1594-3C	$h^{?}$ rae1-167 reb1Δ::kan^{r} cut11^{+}:GFP:ura4^{+} ura4-D18? leu1-32 ade6	This study	Supplementary Fig.2
UKK1591-4C	$h^{?}$ rae1-167 ker1Δ::kan^{r} cut11^{+}:GFP:ura4^{+} ura4-D18? leu1-32 ade6	This study	Supplementary Fig.2
UKK1382-2C	$h^{?}$ rae1-167 rpl501Δ::kan^{r} cut11^{+}:GFP:ura4^{+} ura4-D18 leu1-32 ade6	This study	Supplementary Fig.2
UKK1390-2C	$h^{?}$ rae1-167 rpp102Δ::kan^{r} cut11^{+}:GFP:ura4^{+} ura4 leu1-32 ade6	This study	Supplementary Fig.2
UKK1403-2C	$h^{?}$ rae1-167 rpp201Δ::kan^{r} cut11^{+}:GFP:ura4^{+} ura4-D18 leu1-32 ade6	This study	Supplementary Fig.2
UKK707-1A	$h^{?}$ rae1-167 pabpΔ::kan^{r} cut11^{+}:GFP:ura4^{+} ura4-D18 leu1-32 ade6	This study	Supplementary Fig.2
UKK728-6D	$h^{?}$ rae1-167 pab2Δ::kan^{r} cut11^{+}:GFP:ura4^{+} ura4-D18 leu1-32 ade6	This study	Supplementary Fig.2
UKK1381-4A	$h^{?}$ rae1-167 cip2Δ::kan^{r} cut11^{+}:GFP:ura4^{+} ura4-D18 leu1-32 ade6	This study	Supplementary Fig.2
UKK1375-1B	$h^{?}$ rae1-167 nab3Δ::kan^{r} cut11^{+}:GFP:ura4^{+} ura4-D18 leu1-32 ade6	This study	Supplementary Fig.2
UKK1414-3B	$h^{?}$ rae1-167 SPBC215.06cΔ::kan^{r} cut11^{+}:GFP:ura4^{+} ura4-D18 leu1-32 ade6	This study	Supplementary Fig.2
UKK1396-3C	$h^{?}$ rae1-167 nrp1Δ::kan^{r} cut11^{+}:GFP:ura4^{+} ura4-D18 leu1-32 ade6	This study	Supplementary Fig.2

UKK1626-3A	*h*$^{?}$ *rae1-167 rnc1Δ::kan*^{r} *cut11*$^{+}$*:GFP:ura4*$^{+}$ *ura4-D18? leu1-32 ade6*	This study	Supplementary Fig.2
UKK677-1B	*h*$^{?}$ *rae1-167 cid14Δ::kanr cut11*$^{+}$*:GFP:ura4*$^{+}$ *ura4-D18 leu1-32 ade6*	This study	Supplementary Fig.2
UKK1410-1B	*h*$^{?}$ *rae1-167 nap1Δ::kan*^{r} *cut11*$^{+}$*:GFP:ura4*$^{+}$ *ura4-D18 leu1-32 ade6*	This study	Supplementary Fig.2
UKK592-2D	*h*$^{?}$ *rae1-167 clr3Δ::kan*^{r} *clr4Δ::LEU2 cut11*$^{+}$*:GFP:ura4*$^{+}$ *ura4-D18 leu1-32*	This study	Supplementary Fig.2
UKK729-1B	*h*$^{?}$ *rae1-167 mei4Δ::kan*^{r} *cut11*$^{+}$*:GFP:ura4*$^{+}$ *ura4-D18 leu1-32 ade6*	This study	Supplementary Fig.2
UKK884-2C	*h*$^{?}$ *rae1-167 cut14-208 cut11*$^{+}$*:GFP:ura4*$^{+}$ *ura4-D18 leu1-32*	This study	Supplementary Fig.2
UKK1634-5D	*h*$^{?}$ *rae1-167 dcr1Δ::kan*^{r} *cut11*$^{+}$*:GFP:ura4*$^{+}$ *ura4-D18? leu1-32 ade6*	This study	Supplementary Fig.2
UKK1402-1D	*h*$^{?}$ *rae1-167 hhf1Δ::kan*^{r} *cut11*$^{+}$*:GFP:ura4*$^{+}$ *ura4-D18 leu1-32 ade6*	This study	Supplementary Fig.2
UKK535-1D	*h*$^{?}$ *rae1-167 cds1Δ::kan*^{r} *cut11*$^{+}$*:GFP:ura4*$^{+}$ *ura4-D18 leu1-32 ade6*	This study	Supplementary Fig.2
UKK1661-2D	*h*$^{+}$ *rae1-167 cdr1Δ::ura4*$^{+}$ *cut11*$^{+}$*:mCherry:nat*^{r} *ura4-D18 leu1-32 his2*	This study	Supplementary Fig.2
UKK1666-1A	*h*$^{-}$ *rae1-167 cdr2Δ::ura4*$^{+}$ *cut11*$^{+}$*:mCherry:nat*^{r} *ura4-D18 leu1-32*	This study	Supplementary Fig.2
UKK1601-2D	*h*$^{?}$ *rae1-167 ckb2Δ::kan*^{r} *cut11*$^{+}$*:GFP:ura4*$^{+}$ *ura4-D18? leu1-32 ade6*	This study	Supplementary Fig.2
UKK1615-1B	*h*$^{+}$ *rae1-167 gsk3Δ::ura4*$^{+}$ *cut11*$^{+}$*:mCherry:nat*^{r} *ura4-D18 leu1-32 his2 ade6*	This study	Supplementary Fig.2
UKK1659-2B	*h*$^{+}$ *rae1-167 gsk31Δ::ura4*$^{+}$ *cut11*$^{+}$*:mCherry:nat*^{r} *ura4-D18 leu1-32 his2*	This study	Supplementary Fig.2
UKK1585-7D	*h*$^{-}$ *rae1-167 lsk1Δ::ura4*$^{+}$ *cut11*$^{+}$*:mCherry:nat*^{r} *ura4-D18 leu1-32 ade6*	This study	Supplementary Fig.2
UKK1587-1D	*h*$^{+}$ *rae1-167 lkh1Δ::ura4*$^{+}$ *cut11*$^{+}$*:mCherry:nat*^{r} *ura4-D18 leu1-32 his2*	This study	Supplementary Fig.2
UKK1589-2A	*h*$^{-}$ *rae1-167 mik1Δ::ura4*$^{+}$ *cut11*$^{+}$*:mCherry:nat*^{r} *ura4-D18 leu1-32*	This study	Supplementary Fig.2
UKK1638-4B	*h*$^{?}$ *rae1-167 mug134Δ::kan*^{r} *cut11*$^{+}$*:GFP:ura4*$^{+}$ *ura4-D18? leu1-32 ade6*	This study	Supplementary Fig.2
UKK1663-5C	*h*$^{-}$ *rae1-167 pek1Δ::ura4*$^{+}$ *cut11*$^{+}$*:mCherry:nat*^{r} *ura4-D18 leu1-32*	This study	Supplementary Fig.2
UKK1607-2D	*h*$^{-}$ *rae1-167 pmk1Δ::ura4*$^{+}$ *cut11*$^{+}$*:mCherry:nat*^{r} *ura4-D18 leu1-32*	This study	Supplementary Fig.2
UKK1621-1B	*h*$^{-}$ *rae1-167 ppk6Δ::ura4*$^{+}$ *cut11*$^{+}$*:mCherry:nat*^{r} *ura4-D18 leu1-32*	This study	Supplementary Fig.2
UKK1683-2D	*h*$^{+}$ *rae1-167 ppk14Δ::ura4*$^{+}$ *cut11*$^{+}$*:mCherry:nat*^{r} *ura4-D18 leu1-32 his2*	This study	Supplementary Fig.2
UKK1675-1A	*h*$^{-}$ *rae1-167 ppk15Δ::ura4*$^{+}$ *cut11*$^{+}$*:mCherry:nat*^{r} *ura4-D18 leu1-32*	This study	Supplementary Fig.2
UKK526-1D	*h*$^{?}$ *rae1-167 rad3Δ::kan*^{r} *cut11*$^{+}$*:GFP:ura4*$^{+}$ *ura4-D18 leu1-32 ade6*	This study	Supplementary Fig.2
UKK1608-2D	*h*$^{-}$ *rae1-167 sty1Δ::ura4*$^{+}$ *cut11*$^{+}$*:mCherry:nat*^{r} *ura4-D18 leu1-32*	This study	Supplementary Fig.2
UKK1632-1D	*h*$^{-}$ *rae1-167 spo4Δ::ura4*$^{+}$ *cut11*$^{+}$*:mCherry:nat*^{r} *ura4-D18 leu1-32 ade6*	This study	Supplementary Fig.2
UKK1645-3A	*h*$^{?}$ *rae1-167 srp1Δ::kan*^{r} *cut11*$^{+}$*:GFP:ura4*$^{+}$ *ura4-D18? leu1-32 ade6*	This study	Supplementary Fig.2

UKK1482-5D	*h*$^{?}$ *rae1-167 sck1Δ::kan*^{r} *cut11*$^{+}$*:GFP:ura4*$^{+}$ *ura4-D18? leu1-32 ade6*	This study	Supplementary Fig.2
UKK1653-1C	*h*$^{+}$ *rae1-167 sck2Δ::ura4*$^{+}$ *cut11*$^{+}$*:mCherry:nat*^{r} *ura4-D18 leu1-32 his2*	This study	Supplementary Fig.2
UKK1660-1C	*h*$^{+}$ *rae1-167 slk1Δ::ura4*$^{+}$ *cut11*$^{+}$*:mCherry:nat*^{r} *ura4-D18 leu1-32 his2*	This study	Supplementary Fig.2
UKK1574-1A	*h*$^{+}$ *rae1-167 ssp2Δ::ura4*$^{+}$ *cut11*$^{+}$*:mCherry:nat*^{r} *ura4-D18 leu1-32 his2 ade6*	This study	Supplementary Fig.2
UKK1617-2A	*h*$^{-}$ *rae1-167 ppk21Δ::ura4*$^{+}$ *cut11*$^{+}$*:mCherry:nat*^{r} *ura4-D18 leu1-32*	This study	Supplementary Fig.2
UKK1050-1A	*h*$^{-}$ *lem2*$^{+}$*:GFP:hph*^{r} *leu1-32*	This study	Supplementary Fig. 6a
UKK2018-9C	*h*$^{?}$ *lem2*$^{+}$*:GFP:hph*^{r} *leu1-32 :lem2*$^{+}$*:GFP:hph*^{r}*:leu1*$^{+}$ *cut11*$^{+}$*:mCherry:nat*^{r} *ura4-D18 ade6*	This study	Supplementary Fig. 6a
UKK1883-4B	*h*$^{+}$ *lem2Δ::kan*^{r} *cut11*$^{+}$*:GFP:ura4*$^{+}$ *leu1-32:lnp1*$^{+}$*:leu1*$^{+}$ *ura4-D18 ade6*	This study	Supplementary Fig. 7

sad1.1 mutant strain was provided by the National Bio-Resource Project (NBRP), Japan.
