# Supplementary material for: Nuclear membrane protein Lem2 regulates nuclear size through membrane flow
Source: Nat Commun. 2019 Apr 23;10:1871. doi: 10.1038/s41467-019-09623-x (PMC6478680; doi:10.1038/s41467-019-09623-x)
Supplement: Supplementary file 3 — Description of Additional Supplementary Files [file 41467_2019_9623_MOESM3_ESM.pdf]

Description of Additional Supplementary files

Fine name: Supplementary Movie 1

Description: Timelapse microscopy of cerulenin treated wild type cells displaying mitotic defects. Representative field from timelapse microscopy of wild type cells grown at 32˚C, treated with 10 µg/ml cerulenin. Cell (brightfield, magenta), nuclear envelope (Cut11-GFP, green) maximum intensity projection. Scale bar: 10 µm. Time from 0 to 300 minutes (imaged at five minute intervals) shown (6 frames per second). Cerulenin added at -1 minute.

Fine name: Supplementary Movie 2

Description: Timelapse microscopy of untreated wild type cells. Representative field from timelapse microscopy of wild type cells grown at 32˚C, untreated. Cell (brightfield, magenta), nuclear envelope (Cut11-GFP, green) maximum intensity projection. Scale bar: 10 µm. Time from 0 to 300 minutes (imaged at five minute intervals) shown (6 frames per second). EtOH control for drug treatment added at -1 minute.

Fine name: Supplementary Movie 3

Description: Timelapse microscopy of cerulenin treated lem2Δ cells displaying mitotic defects and interphase nuclear shrinkage. Representative field from timelapse microscopy of lem2Δ cells grown at 32˚C, treated with 10 µg/ml cerulenin. Cell (brightfield, magenta), nclear envelope (Cut11-GFP, green) maximum intensity projection. Scale bar: 10 µm. Time from 0 to 300 minutes (imaged at five minute intervals) shown (6 frames per second). Cerulenin added at -1 minute.

Fine name: Supplementary Movie 4

Description: Timelapse microscopy of untreated lem2Δ cells. Representative field from timelapse microscopy of lem2Δ cells grown at 32˚C, untreated. Cell (brightfield, magenta), nuclear envelope (Cut11-GFP, green) maximum intensity projection. Scale bar: 10 µm. Time from 0 to 300 minutes (imaged at five minute intervals) shown (6 frames per second). EtOH control for drug treatment added at -1 minute.

Fine name: Supplementary Movie 5

Description: Timelapse microscopy of cerulenin treated wild type cells. Representative field from timelapse microscopy of wild type cells grown at 32˚C, treated with 10 µg/ml cerulenin. Nuclear envelope (Cut11-GFP, grayscale) maximum intensity projection Scale bar: 10 µm. Time from 0 to 150 minutes (imaged at five minute intervals) shown (6 frames per second). Cerulenin added at -2 minutes.

Fine name: Supplementary Movie 6

Description: Timelapse microscopy of untreated wild type cells. Representative field from timelapse microscopy of wild type cells grown at 32˚C, untreated. Nuclear envelope (Cut11-GFP, grayscale) maximum intensity projection Scale bar: 10 µm. Time from 0 to 150 minutes (imaged at five minute intervals) shown (6 frames per second). EtOH control for drug treatment added at -2 minutes.

Fine name: Supplementary Movie 7

Description: Timelapse microscopy of cerulenin treated lem2Δ cells displaying nuclear shape changes and membrane blebbing. Representative field from timelapse microscopy of lem2Δ cells grown at 32˚C, treated with 10 µg/ml cerulenin. Nuclear envelope (Cut11-GFP, grayscale) maximum intensity projection Scale bar: 10 µm. Time from 0 to 150 minutes (imaged at five minute intervals) shown (6 frames per second). Cerulenin added at -2 minutes.

Fine name: Supplementary Movie 8

Description: Timelapse microscopy of untreated lem2Δ cells. Representative field from timelapse microscopy of lem2Δ cells grown at 32˚C, untreated. Nuclear envelope (Cut11-GFP, grayscale) maximum intensity projection Scale bar: 10 µm. Time from 0 to 150 minutes (imaged at five minute intervals) shown (6 frames per second). EtOH control for drug treatment added at -2 minutes.
